# Nuclear Inositides and Inositide-Dependent Signaling Pathways in Myelodysplastic Syndromes

**DOI:** 10.3390/cells9030697

**Published:** 2020-03-12

**Authors:** Jie Xian, Eric Owusu Obeng, Stefano Ratti, Isabella Rusciano, Maria Vittoria Marvi, Antonietta Fazio, Alessia De Stefano, Sara Mongiorgi, Alessandra Cappellini, Giulia Ramazzotti, Lucia Manzoli, Lucio Cocco, Matilde Yung Follo

**Affiliations:** Cellular Signalling Laboratory, Department of Biomedical and Neuromotor Sciences (DIBINEM), University of Bologna, 40126 Bologna, Italy; jie.xian2@unibo.it (J.X.); eric.owusuobeng2@unibo.it (E.O.O.); stefano.ratti@unibo.it (S.R.); isabella.rusciano3@unibo.it (I.R.); mariavittoria.marvi2@unibo.it (M.V.M.); antonietta.fazio2@unibo.it (A.F.); alessia.destefano@studio.unibo.it (A.D.S.); s.mongiorgi@unibo.it (S.M.); alessandra.cappellini@unibo.it (A.C.); giulia.ramazzotti@unibo.it (G.R.); lucia.manzoli@unibo.it (L.M.); matilde.follo@unibo.it (M.Y.F.)

**Keywords:** nucleus, speckles, phospholipases, PI3K/Akt/mTOR, PLCβ1, myelodysplastic syndromes, nuclear inositides

## Abstract

Myelodysplastic syndromes (MDS) are a heterogeneous group of hematological malignancies characterized by peripheral blood cytopenia and abnormal myeloproliferation, as well as a variable risk of evolution into acute myeloid leukemia (AML). The nucleus is a highly organized organelle with several distinct domains where nuclear inositides localize to mediate essential cellular events. Nuclear inositides play a critical role in the modulation of erythropoiesis or myelopoiesis. Here, we briefly review the nuclear structure, the localization of inositides and their metabolic enzymes in subnuclear compartments, and the molecular aspects of nuclear inositides in MDS.

## 1. Introduction

**Figure 1:**
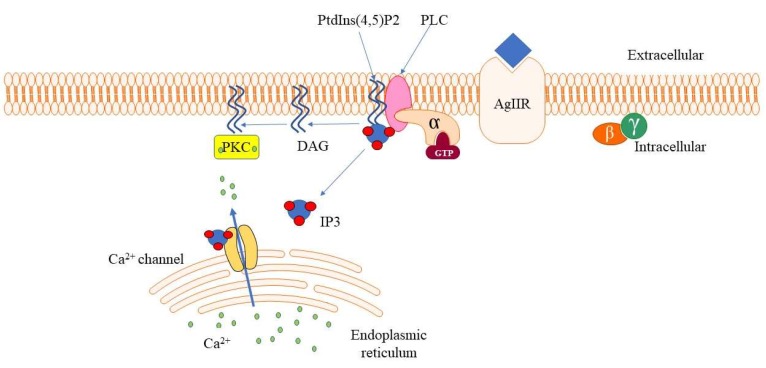
A cartoon representation of phospholipase beta (PLCβ) signaling. PLCβ hydrolyzes membrane-bound PtdIns(4,5)P2 to inositol 1,4,5-trisphophate (IP3) and diacylglycerol (DAG), which are important second messengers in the downstream signaling pathway, regulating Ca^2+^ mobilization and protein kinase C (PKC) activation.

Phosphoinositides (PIs) are inositol phospholipids constituted of hydrophilic inositol groups linked to two fatty chains, which are involved in several signaling pathways. PIs represent the most frequently studied phospholipids. They are composed of the precursor phosphatidylinositol (PtdIns) and its phosphorylated derivatives of seven members [1]. PIs play several pivotal roles in cell proliferation, cell differentiation, and gene expression. The kinases and phosphatases related to the PI pool are present at both the plasma membrane and nuclear level, within several distinct compartments of the nucleus, like the nuclear speckles [2]. Phosphoinositide-specific phospholipases (PLCs) are a group of inositide-dependent enzymes that cleave phosphatidylinositol 4,5-biphosphate (PtdIns(4,5)P2) to inositol 1,4,5-trisphophate (IP3) and diacylglycerol (DAG). These are key second messengers that induce or inhibit cell proliferation, cell apoptosis, activation of immune, cells and stem cell differentiation via intracellular release of calcium ions and activation of protein kinase C (PKC), respectively [3,4] (Figure 1).

A number of PLC isoforms are found in the nucleus together with their substrate PIs [5,6,7]. It is interesting to take into account that nuclear PLC, namely PLCβ1, is regulated differently than the one at the plasma membrane. Moreover, it has been recently shown that PI receptors occur in the nucleus, where they mediate the binding interactions between effector proteins and nuclear PIs [8,9,10]. The phosphatidylinositol-3-kinase (PI3K)/Akt/mTOR signaling pathway plays an important role in the control of several cellular processes, such as cell growth, proliferation, survival, and neoplastic transformation [11,12]. Several stimuli, including a range of growth factors and mitogens, activate cell surface tyrosine kinase receptors, which in turn determine the activation of PI3K. For further activation, Akt is phosphorylated by mammalian target of rapamycin (mTOR) to regulate cell metabolism and differentiation [13,14,15]. In addition, the PI3K/Akt/mTOR pathway has some overlapped functions with PLCs and PKC [16].

Myelodysplastic syndromes (MDS) are a heterogeneous group of hematological malignancies characterized by peripheral blood cytopenia and abnormal myeloproliferation, with a variable risk of evolution into acute myeloid leukemia (AML) [17]. Although the therapy regimen for MDS patients has seen improvements in recent years, there are no therapies to quickly eradicate the disease, except allogeneic stem cell transplantation [18]. The first line of treatment for MDS is an epigenetic therapy which involves the use of demethylating agents, administered alone or in combination with other drugs. However, MDS patients at higher risk of AML evolution can become resistant to this therapy [19]. Recently, a molecular study has linked a few inositide-related genes to the lack of response to epigenetic therapy [20]. However, the mechanisms of appropriate inositide-dependent interactions and regulatory signals that alter several critical cellular events implicated in MDS, such as cell proliferation or apoptosis, are still not fully understood [21]. Therefore, a better comprehension of inositide signaling in MDS could be helpful. Here, we briefly describe the nuclear architecture and nuclear inositide metabolism while establishing a link between nuclear inositide-dependent signaling and deregulated pathways in MDS.

## 2. Nuclear Structure and Nuclear Inositides

**Figure 2:**
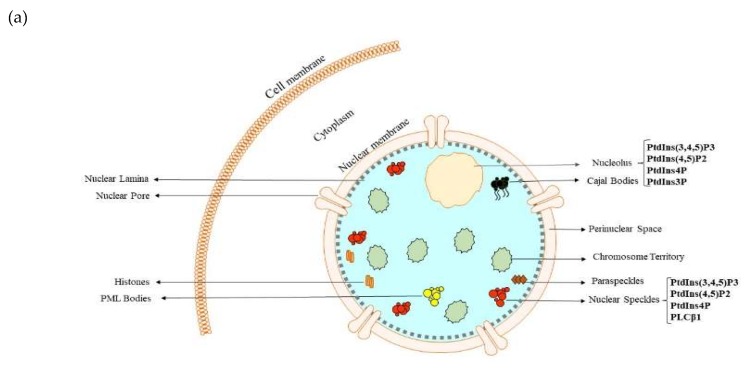

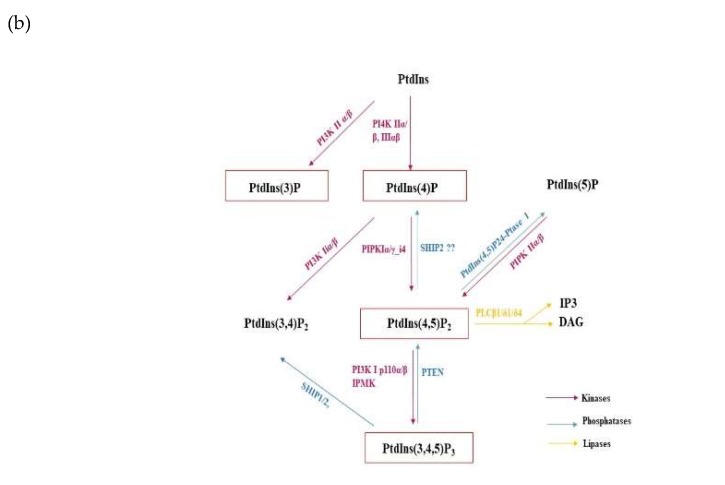
Nuclear subcompartments and nuclear inositide signaling. (**a**) A cartoon representation of the mammalian nucleus and some of its identified membraneless subcompartmental domains. Numerous inositides localize within these domains in the nucleus, while regulating gene expression, i.e., speckles; (**b**) Schematic representation of nuclear phosphoinositide (PI) signaling involving nuclear PIs and their nuclear metabolic enzymes.

The nucleus is a double membrane-bound eukaryotic cell organelle with a diameter ranging from 5 to 10 µm that houses the genome of eukaryotes and consists of several subcompartments. [22]. It was one of the first intracellular organelles to be discovered, but comprehension of its overall structure at the subnuclear level and functions are still not clear. Many studies have demonstrated that the nucleus is highly organized with various membraneless and extremely dynamic subnuclear compartments [22,23,24]. Also, chromosomes are nonrandomly distributed in the nucleus, as they localize across distinct regions, called chromosome territories, and interact with various subnuclear compartments such as nuclear speckles, nucleoli, paraspeckles, cajal bodies, nuclear pore complexes, nuclear lamina, and promyelocytic leukemia bodies (Figure 2) [22,25]. This suggests that nuclear localization of key molecules is not random and several incoming reports support the notion that gene expression is influenced by nuclear positioning [25,26,27]. Supporting this notion are evidences of the existence of important players and dynamically exchanging proteins capable of regulating gene expression in each of the subnuclear compartments [22,24,25]. For example, nuclear speckles, also called interchromatin granule clusters or the splicing factor domain, are membraneless subnuclear compartments located in the interchromatin regions of the nucleus [24]. They are frequently presented as 20 to 50 punctate, irregular structures with varying sizes and shapes under the fluorescence microscope, whereas they appear as clusters of interchromatin granules under the electron microscope. Nuclear speckles are reservoirs of pre-messenger RNA splicing factors and other transcription regulatory proteins which are frequently recruited to active transcription sites [24,25], which is why genes localizing to nuclear speckles are involved in gene expression.

Since the 1950s, when the first evidence of PIs was demonstrated [28], most research on the PI cycle focused on the functional mechanisms of cytosolic membrane-associated PIs, until observations of the presence of a phospholipid content in the nuclear chromatin were reported [29,30]. This was an intriguing period for lipid research and it inspired a massive interest in nuclear research, leading to the discovery, in 1983, of phosphatidylinositol phosphate kinase activity in the nucleus [31] and, subsequently, a distinct PI cycle in the nucleus [32,33,34].

Although PIs represent just a tiny fraction of the total lipid content at the plasma membrane, there is strong evidence from lipidomic mass spectrometry of a high amount of PIs in the nucleus [35]. Nuclear PIs are not restricted just to membrane structures, but they can also localize to non-membranous structures [36]. Until now, a plethora of studies have confirmed the existence of PIs and their metabolizing enzymes, i.e., lipases, kinases, and phosphatases, in several subnuclear compartments. This generates a distinct PI pool capable of regulating several essential nuclear processes, such as chromatin remodeling, DNA repair, RNA processing, and gene transcription [1,26,37]. The ability of PI metabolizing enzymes to localize within the same nuclear subcompartments as their respective substrate PI or product confirms their direct involvement in nuclear PI metabolism (Figure 2).

A lot of mystery remains associated with nuclear PIs. For example, in which form do nuclear PIs exist in the nucleus? Do they generate distinct functions related to their distribution at different nuclear compartments? Are they only transported to the nucleus upon response to certain stimuli or are they synthesized in the nuclei? How do they interact with effector or target proteins to regulate nuclear functions? Answering these essential questions is paramount in deciphering the key functional mechanisms of nuclear PIs [1,26,35]. However, detection of PIs, especially nuclear PIs, remains one of the biggest challenges in this field. Current methods of detecting PI in vivo utilize light and electron microscope to visualize antibodies and PI-binding domains that target specific PIs [38]. Using different advanced techniques, all PIs, except PtdIns(3,5)P2, have been demonstrated to localize to the nucleus (Table 1) [26,27], being distributed across multiple nuclear sites such as speckles and nucleoli [36].

## 3. Nuclear Processes Regulated by Nuclear PIs

Since the nucleus is the genetic powerhouse of cells, it is easy to believe that nuclear localization of PIs and their metabolizing enzymes allows them to regulate gene and protein expression. Jungmichel et al. showed by qualitative mass spectrometry analysis that nuclear PIs are bound to over 120 nuclear proteins expressing different affinities for nuclear PIs [69]. Their report suggests that nuclear PIs mediate numerous nuclear processes, as they are bound to different nuclear proteins. These functions are extensively described, but we briefly review a few of these processes below.

### 3.1. Gene Expression

Gene expression in eukaryotes involves a series of tightly regulated nuclear events spanning from RNA processing to RNA export into the cytoplasm. Precursor RNA undergoes further processing, such as 5′ capping, 3′ cleavage, addition of the Poly A tail (polyadenylation), and RNA splicing, before mature RNA is transported into the cytoplasm. There is increasing amount of evidences linking the regulation of these processes to nuclear PIs. For instance, Osborne et al. demonstrated in their in vitro studies the involvement of nuclear PtdIns(4,5)P2 in gene transcription and pre-mRNA splicing. They revealed an interaction between nuclear PtdIns(4,5)P2, the hyperphosphorylated form of the large unit of RNA polymerase II and the nuclear speckle protein SC-35. Indeed, through immunodepletion, PIs inhibited pre-mRNA splicing [42]. In addition, from a yeast-two hybrid screen performed by Mellman et al. to identify nuclear PIPKIα interacting proteins, an interaction between nuclear PtdIns(4,5)P2 and a poly (A) polymerase called Star-PAP (speckle targeted PIPKIα regulated-poly (A) polymerase) was observed [70]. Through this interaction, nuclear PtdIns(4,5)P2 stimulated the initiation and elongation steps in polyadenylation. Furthermore, IPMK (human inositol polyphosphate multikinase) was also reported to mediate a transcript-selective nuclear mRNA transport into the cytoplasm by generating PtdIns(3,4,5)P3 [59].

### 3.2. Chromatin Remodeling

Eukaryotic DNA is highly condensed in the nucleus and DNA is tightly wound around histone proteins to form the chromatic complex. Gene expression is regulated by chromatin remodeling. Numerous studies have pointed out the effects of some PIs on chromatin opening. Typical examples are the involvement of PtdIns(4,5)P2 and PtdIns5P. Nuclear PtdIns(4,5)P2 has been shown to regulate chromatin by interacting with the SW1/SNF-like/BAF (BRM-associated factors) chromatin remodeling complex, through its ATPase subcomponent BRG1 [71]. In addition, nuclear PtdIns(4,5)P2 directly communicates with H1 and H3 histone proteins. Several studies reported the ability of H1 to inhibit RNA polymerase II activity during transcription and, interestingly, this is partially reversed upon adding back PtdIns(4,5)P2 [72,73]. Nuclear PtdIns5P regulates chromatin remodeling by interacting with the histone code reader associated with the chromatin complex ING2 (inhibitor of growth protein 2), which is a subunit of the HDAC1 complex (Sin3a-histone deacetylase 1). In particular, PtdIns5P modulates the binding of ING2 to chromatin and also participates in epigenetic gene expression and DNA damage [37,74].

### 3.3. Cell Survival

PtdIns(3,4,5)P3 is involved in major signaling axes implicated in cell survival by promoting the expression of anti-apoptotic signals [37]. At the nuclear level, PtdIns(3,4,5)P3 is stimulated by NGF (nerve growth factor) to mediate cell survival. It interacts with nucleophosmin (B23) to form the PtdIns(3,4,5)P3-B23 complex, which prevents DNA fragmentation by inhibiting the nuclease activity of CAD (caspase-activated DNase), as well as protecting cells from proteolytic cleavage by interacting with nuclear Akt (Protein kinase B) [75,76].

Together, these provided evidences underscore the relevance of understanding the signaling cascades associated with nuclear PIs and their effector proteins. Extending this knowledge to pathologies, such as MDS, could be beneficial for therapeutic innovations.

## 4. Nuclear Inositide Dependent Signaling in MDS

### 4.1. PLCs

Since the first evidence of the existence of PLCs in 1953 [77], 13 mammalian PLC isozymes have been identified so far and they are divided into six subfamilies (β, γ, ε, δ, ζ, η). Interestingly, all PLC isozymes show highly conserved domains (X and Y), as well as unique mingled domains (C2 domain, the EF-hand motif, and the pleckstrin homology domain) [78]. The activation and regulation of PLC isozymes differ in their peculiar subtype structure. Usually, PLCβ enzymes are activated by G protein-coupled receptors (GPCRs), while PLCγ subtypes are linked to the activation by receptor tyrosine kinase (RTK) via SH2 domain-phospho-tyrosine interaction [3]. Moreover, there are several reports of the involvement of specific PLCs in a number of disorders [79].

#### 4.1.1. PLCβ1

**Figure 3:**
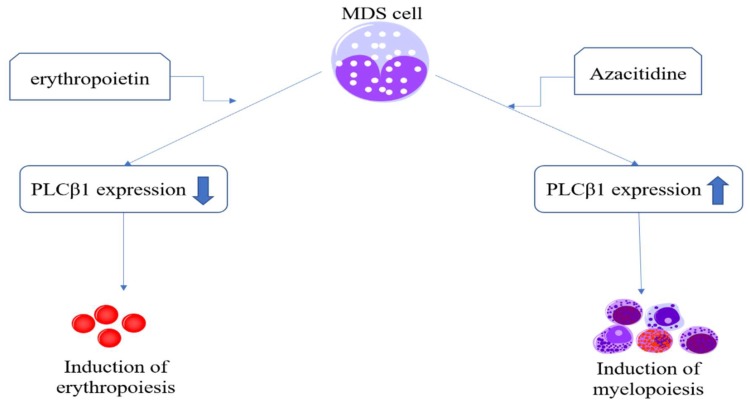
Nuclear PLCβ1 expression is critically associated with myelopoiesis and erythropoiesis of myelodysplastic syndromes (MDS) cells. Induced by demethylating agents, such as azacitidine, nuclear PLCβ1 shows a higher expression to promote myelopoiesis of MDS cells. On the contrary, when treated with erythropoietin, MDS cells downregulate nuclear PLCβ1 expression.

As a key inositide-dependent enzyme, PLCβ1 regulates several critical cellular processes, both at the nuclear and cytoplasmic levels. PLCβ1 is involved in both G1/S and G2/M cell cycle phases by modulating different proteins, such as cyclin D3, cyclin E, and lamin B1 [80,81,82,83]. In recent years, azacitidine (AZA) and decitabine demethylating agents have been used to treat MDS patients [17,84]. Interestingly, many studies have confirmed that PLCβ1 is a molecular target for AZA [85,86,87,88], inducing an increase of myeloid differentiation (Figure 3) [89]. PLCβ1 can be involved in AZA-induced myeloid differentiation through the recruitment of the myeloid zinc finger (MZF-1) on the promoter of PLCβ1, as it is specially recruited in MDS patients responding to AZA therapies [90]. The level of PLCβ1 RNA expression has also been recognized as an important factor to anticipate MDS patients’ clinical outcome during hypomethylating therapies [90,91].

Recent studies have also demonstrated the importance of nuclear inositides in MDS patients treated with AZA and lenalidomide, the latter being a drug that is particularly effective in MDS patients showing a specific deletion of chromosome 5q [del(5q)]. These patients are usually at lower risk of AML evolution, but are characterized by ineffective erythropoiesis. Little is known about the molecular effect of lenalidomide, but, currently, the best hypothesis is that it suppresses the del(5q) clone and restores a normal erythropoiesis [20,92,93]. One study analyzed the effect of lenalidomide on 16 patients with low-risk del(5q) MDS, as well as del(5q) and non-del(5q) hematopoietic cell lines, focusing on erythropoiesis, cell cycle, and PLCβ1/PKCα signaling [94]. This study revealed PLCβ1 localization within the cytoplasm of the del(5q) cells, whereas, in the same subpopulation, PKCα, a serine/threonine kinase downstream of PLCβ1, translocated to the nucleus. All these evidences reveal the role of PLCβ1/PKCα signaling in erythroid differentiation on del(5q) low-risk MDS patients responding to lenalidomide, and thus opening the way to innovative targeted therapies.

Indeed, in recent years, erythropoiesis-stimulating agents (ESAs) were used as first-line therapy in most patients with lower-risk non-del(5q) MDS, mainly with the aim of managing anemia and the prevention of transfusion-related complications which is associate with secondary organ damage, decreased survival, and leukemic progression [95,96]. Thus, ESAs aim to correct anemia by stimulating proliferation and differentiation of normal residual erythroid progenitors. Among ESAs, erythropoietin and mimetics, such as darbepoetin-EPO’s glycosylated form, are the first choice. However, clinical studies have shown that approximately 30% of MDS patients are resistant towards ESAs right from the beginning, as determined after 8–12 weeks of treatment, or they eventually lose response over time [97]. Even though many molecular studies have proposed several different assumptions in recent years [98,99], the exact molecular mechanisms of ESAs in low-risk MDS patients and their clinical resistance remain poorly understood. Our previous studies demonstrated that PLCβ1 could be a negative regulator of erythropoiesis in MDS, and that an inverse correlation between PLCβ1 and Akt expression could be observed in high-risk MDS patients [85]. The increased expression of PLCβ1 could indeed induce the downregulation of phosphorylated Akt, whose activation could lead to a decrease in apoptosis while increasing survival of MDS cells. Moreover, in low-risk MDS patients responding to ESAs therapies, namely erythropoietin (EPO) [86], EPO was associated with several inositide signaling pathways, such as PI3K/Akt/PLCγ1, resulting in apoptosis and a low proliferation rate of MDS cells.

#### 4.1.2. PLCγ1

Similar to like PLCβ1, PLCγ1 is an inositide-dependent key metabolizing enzyme which is involved in an enzymatic reaction that produces IP3 and DAG [3,83]. Although both enzymes share several common features in molecular structure and function, PLCγ1 still has some unique peculiarities in its activation and downstream signaling. PLCγ1 activity is regulated by PI3K through the interaction of the PI3K product PtdIns(3,4,5)P3 and PLCγ1 PH domain. PLCγ1 activates mitogen-mediated signaling events through second messengers, leading to gene expression changes via PKC [4,100].

Recent studies have demonstrated that PLCγ1 is associated with hematopoiesis in vivo [101] and also with the differentiation of embryonic stem cells into erythrocytes and monocytes/macrophages in vitro [102]. Other studies have shown that the PLCγ1-induced Raf1 signaling could be inhibited by the PI3K/Akt1 pathway [103,104] and, more importantly, genetic mutations in PLCγ1 gene (y10 allele) could completely disrupt the aortic blood flow and hematopoietic stem/progenitor cells (HSPC) formation [4]. On the basis of these evidences, it is obvious that PLCγ1 plays a critical role in mammalian hematopoiesis and vasculogenesis, which are also closely linked to MDS clinical characterization.

ESAs play an important role in reversing anemia also through PLCγ1 signaling pathways. One of our previous studies confirmed that PLCγ1 expression is high in EPO responder low-risk MDS patients, whereas it is not significantly affected in patients refractory to EPO [105]. Binding of EPO to its EPO receptor is required for erythropoiesis. At a molecular level, EPO activation could activate PLCγ1 [106]. Another study also verified that mH2A2, a downstream effector of PLCγ1, plays the same function on the regulation of erythroid differentiation, indicating that PLCγ1 and its downstream target mH2A2 signaling could be a ”non-canonical” EPO signaling pathway essential for erythroid differentiation [107].

Nuclear and cytoplasmic PLCγ1 are also implicated in the regulation of the lymphoid lineage, particularly in T cells, but with increasing studies on early B cells [108,109], PLCγ1 seems to be essential for the regulation of T cells activation and differentiation, through the classical T cell receptor (TCR) signaling pathway. In addition, PLCγ1 is also involved in the context of signaling initiated by both growth factors and non-receptor tyrosine kinases, such as epidermal growth factor and hepatocyte growth factor [89,110,111]. This is also supported by another recent study which demonstrated that, along with transforming growth factor beta (TGF-β1), PLCγ1 acts as an immune suppressor by regulating immune cells differentiation and tolerance induction, suggesting the modulation of the PLCγ1/TGF-β1 pathway in T cell differentiation and immune activation [112]. Moreover, not only PLCγ1 but also its downstream products, IP3 and DAG, are associated with several different essential signaling pathways, for example Ras, ERK, and NFAT [113,114]. Interestingly, a specific gene mutation of the catalytic domain of PLCγ1 was observed in cutaneous T cell lymphomas [115]. A similar function could also be observed in the regulation of pre-B-cell differentiation, as the reduced expression of PLCγ1 could impede early B-cell development at the pro-B/pre-B-cell transition [116].

### 4.2. PI3K/Akt/mTOR

PI3K is a serine/threonine kinase that phosphorylates PtdIns(4,5)P2 to PtdIns(3,4,5)P3, which, in turn, can be a docking site for other downstream proteins [117]. Akt can be deactivated by the PTEN phosphatase. In particular, PTEN converts PtdIns(3,4,5)P3 into PtdIns(4,5)P2, directly reversing the effects of PI3K. Thus, PTEN inactivation leads to PtdIns(3,4,5)P3 accumulation and, consequently, to the hyperactivation of Akt [13,14,15]. The PI3K/Akt pathway is involved with mTOR signaling, which is downstream and represents one of the frequently deregulated pathways in cancer cells, and one of the most important targets of new cancer therapies [118]. Following Akt activation, phosphorylated mTOR activates its downstream pathways to regulate DNA repair and transcription, RNA dynamics, and protein synthesis. The structure of mTOR is peculiar, as it is constituted by two molecular complexes, mTORC1 and mTORC2. On the one hand, phosphorylated mTORC2 can activate other downstream targets that can induce a negative feedback activation of Akt, thus, inducing a loop on cell growth and protein synthesis [119,120]. On the other hand, mTORC1 is particularly associated with autophagy [121]. Autophagy has been recognized to be induced during oxidative stress where there are elevated intracellular levels of reactive oxygen species (ROS). Interestingly, ROS is associated with inositide signaling in MDS pathogenesis [122,123].

Deregulation of PI3K or Akt genes and the upstream molecular targets of the PI3K/Akt/mTOR pathway are also detectable in more than 60% AML cases. Meanwhile, although mTOR mutations are rarely observed in tumor cells, its gene loss is always associated with cancerogenesis and regarded as a potential target for strategic therapies [124].

## 5. Conclusions

The nucleus is a highly organized organelle with several distinct compartments where inositides and their metabolic enzymes localize to mediate essential cellular events. Nuclear inositides and some specific enzymes, such as PLCs, are becoming new molecular targets in MDS for many cellular processes, such as cell growth, proliferation, survival, and cellular transformation. As shown here, PLCβ1 induced myeloid specific differentiation and also inhibited erythroid differentiation in MDS. Along with PLCβ1, PLCγ1 signal transduction pathways are interconnected with the activation of the PI3K/Akt/mTOR axis, which is specifically associated with leukemogenesis, and therefore is activated during MDS progression to AML. PLCγ1 could also be extremely important during the early phases of erythroid differentiation, as its specific induction in this lineage could be essential to induce a correct erythropoiesis.

All in all, nuclear inositides could become innovative therapeutic targets or new therapeutic indicators useful to test the efficacy of the treatment in MDS patients.

## Figures and Tables

**Table 1:** Subcompartmental localization of nuclear PIs and their metabolic enzymes.

Phosphoinositides	Nuclear Localization
PtdIns(3,4,5)P3	Matrix [39], nucleoplasm [40], speckles [41], Nucleoli [40]
PtdIns(4,5)P2	Speckles, nucleoli [42], nucleoplasm [38], nuclear lipid islets [43]
PtdIns (3,4)P2	Speckles [41], nuclear membrane [44]
PtdIns5P	Chromatin and matrix [45]
PtdIns4P	Nucleoli [38], speckles [38], chromatin [35]
PtdIns3P	Nucleoli [35] matrix [46]
**PI Dependent Metabolizing Enzymes**	
**Nuclear Phospholipases**	
PLCβ1	Speckles [6], matrix [5]
PLCδ1	Matrix [47]
PLCδ4	Nucleus [7]
PLCε	Perinuclear space [48]
**Nuclear Phosphatases**	
PTEN	Nucleoli [49], chromatin [50]
INPP5K/SKIP	Speckles [51]
SHIP1	Nucleoli [52]
SHIP2	Speckles [41]
Type I PtdIns(4,5)P2 4-phosphatase	Nucleus [53]
**Nuclear Kinases**	
DGKθ	Speckles [54]
DGK isoforms: α, ζ	Matrix [55,56]
PKC isoforms: α, βII, δ, η	Nucleus [57,58]
IPMK	Nucleus [59]
PIPKIα	Speckles [60], nucleoplasm [60], matrix [61]
PIPKIγ_i4	Speckles [62], matrix [61]
PIPKIIα	Speckles [63], matrix [61]
PIPKIIβ	Speckles [63], matrix [61]
PI3K p110β	Nucleus [64]
PI3K IIα	Speckles [65]
PI3K IIβ	Matrix [46,66]
PI4KIIα	Nucleus [67]
PI4KIIβ	Speckles [68]
PI4KIIIα	Nucleoplasm and Nucleoli [67]
PI4KIIIβ	Nuclear pore [49]

Abbreviations: PtdIns, phosphatidylinositol; PLC, phospholipase C; PTEN, phosphatase and tensin homolog deleted on chromosome 10; INPP5K, inositol polyphosphate 5 phosphatase K; SKIP, skeletal muscle and kidney enriched inositol phosphatase; SHIP, src homology 2 (SH2) domain containing inositol phosphatase; DGK, diacylglycerol kinase; PKC, protein kinase C, IPMK, inositol polyphosphate multikinase; PIPK, phosphatidylinositol phosphate kinase; PI3K, phosphatidylinositol-3-kinase; PI4K, phosphatidylinositol 4-kinase.
